# A Study of Null Broadening Algorithms for Navigation Receivers in Highly Dynamic Scenarios

**DOI:** 10.3390/s25051499

**Published:** 2025-02-28

**Authors:** Yuanfa Ji, Tao He, Yu Chen, Chenggan Wen, Xiyan Sun

**Affiliations:** 1 Information and Communication School, Guilin University of Electronic Technology, Guilin 541004, China; jiyuanfa@163.com (Y.J.); he_tao1204@163.com (T.H.); 2 Guangxi Key Laboratory of Precision Navigation Technology and Application, Guilin University of Electronic Technology, Guilin 541004, China; 3 College of Semiconductors (College of Integrated Circuits), Hunan University, Changsha 410082, China; wenchenggan@hnu.edu.cn

**Keywords:** GNSS, anti-jamming, highly dynamic, beam null broadening

## Abstract

Due to the narrow nulls formed by the Power Inversion (PI) algorithm, it fails to suppress jamming signals in highly dynamic scenarios effectively. This paper proposes a null broadening algorithm based on eigenvalue sorting. Unlike other algorithms, this one does not require prior knowledge of the direction of jamming. It is based on the Covariance Matrix Taper (CMT) algorithm, which orders the eigenvalues of the sampling covariance matrix. The sample covariance matrix’s eigenvalues are sorted to provide new sample data, and the rebuilt covariance matrix is then averaged forward and backward. The experimental results demonstrate that the proposed algorithm can effectively broaden the null. Compared with the CMT algorithm, the null in the jamming direction is, on average, approximately 22 dB deeper under the experimental conditions, and the gain in the direction of the sound signal is increased by around 15 dB. Moreover, the signal can be successfully acquired even when the input jamming-to-signal ratio (ISR) is relatively low. When there is a deviation in the jamming direction, the proposed algorithm demonstrates robust null broadening performance even with a small number of snapshots. The output SINR of the proposed algorithm exhibits a nearly linear relationship with the input SNR.

## 1. Introduction

With the advancement of society, Global Navigation Satellite Systems (GNSSs) have become indispensable in both military and civilian applications [1]. Due to the significant distance between satellites and ground-based receivers, the signal power received by ground receivers is extremely weak [2]. In contrast, jamming sources are much closer to ground receivers than satellites, resulting in jamming signals with power levels significantly higher than those of satellite signals. Consequently, receivers are highly susceptible to jamming, which can lead to signal loss or failure in acquisition [3,4]. To address this issue, the concept of array-based anti-jamming techniques has been introduced into the field of navigation. This approach is based on an optimal criterion, where an array anti-jamming algorithm is used to generate a weight vector, which in turn controls the formation of the beam. By setting nulls in the direction of the jamming source through the formed beam, the jamming signals can be suppressed [5]. However, when the receiver is in a high-speed motion state, the relative velocity between the navigation receiver and the jamming source increases sharply, causing the jamming direction to change continuously [6]. Since the nulls formed by the PI algorithm are very narrow, the direction of the nulls does not align with the jamming direction, making this algorithm unsuitable for highly dynamic environments. Research into anti-jamming algorithms for navigation receivers in highly dynamic environments is of significant practical importance, and beam null broadening technology has become a key technique for solving the anti-jamming challenges in highly dynamic receivers [7]. CMT null broadening algorithm, as a representative method, can broaden nulls but at the cost of reducing null depth [8,9]. Reference [10] employs a Gaussian distribution model to assume variations in the jamming direction for null broadening. Building upon [10], Reference [11] utilizes projection transformations to achieve deeper broadened nulls, yet its anti-jamming performance remains suboptimal under low signal-to-noise ratio (SNR) conditions. Reference [12] proposes a method that reconstructs the INC (Interference-plus-Noise Covariance) matrix by focusing on the desired signal’s arrival region combined with spatial spectra, removing the desired signal from the covariance matrix to achieve null broadening. However, this approach requires prior knowledge of the desired signal’s direction of arrival.

To address the above-mentioned issues, this paper proposes a null broadening algorithm based on eigenvalue sorting. The algorithm first sorts the eigenvalues of the sample covariance matrix and reconstructs it to enhance the contribution of jamming signals, thereby creating deeper nulls. Subsequently, the CMT broadening algorithm is applied to expand the nulls further. This combined approach allows the receiver to more effectively suppress rapidly changing jamming sources, significantly improving the array’s output SINR and ensuring the effective acquisition of the desired signals. Furthermore, the proposed algorithm integrates the PI algorithm and, specifically addressing the low-power characteristics of navigation satellite signals, combines null broadening techniques with anti-jamming scenarios in satellite navigation systems.

The remainder of this paper is organized as follows: Section 2 provides a detailed derivation of the signal model and defines the highly dynamic scenario. Section 3 introduces the PI algorithm, the CMT algorithm, and the proposed algorithm. In Section 4, the experimental results are presented and analyzed. Section 5 concludes the study and discusses potential directions for future research.

## 2. Modeling of Array Received Signals in Highly Dynamic Scenarios

### 2.1. Definition of a Highly Dynamic Scenario

NASA’s Jet Propulsion Laboratory (JPL) defines two highly dynamic scenarios: the first is a sustained 70 g/s jerk for 1 s; the second is a sustained 100 g/s jerk for 0.5 s, and then a 50 g acceleration maintained for the next 2 s, followed by a sustained −100 g/s acceleration for the next 0.5 s. 

### 2.2. Signal Model

**Figure 1:**
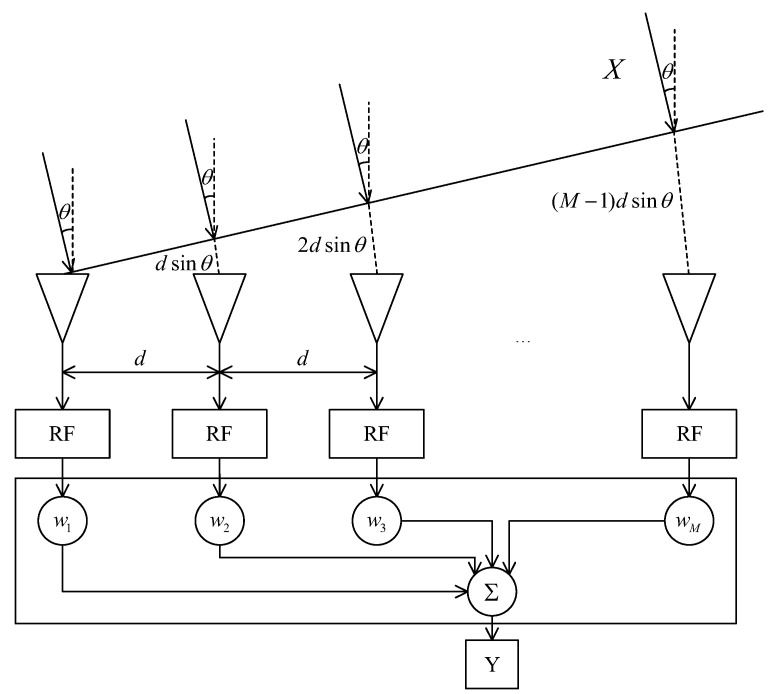
Uniform linear array (ULA) model.

A uniform line array is a one-dimensional array model, and the azimuth angle θ is commonly used to characterize the direction of the signal source’s baud on the antenna array. Assuming that the array is an M-element equidistant line array, the spacing between the array elements is d, the carrier wavelength of the received signal is λ, and the azimuth angle θ represents the angle between the incident signal and the normal of the array, and its value domain is $\left[-\frac{\pi }{2},\frac{\pi }{2}\right]$. The uniform line array model is shown in Figure 1, and the origin of coordinates is set in the position of the leftmost element of the array.

The wave range and phase difference of the m-th array element in comparison to the reference array element are, respectively:

(1) $$\Delta l_{m}=(m-1)d\sin\theta$$

(2) $$\Delta \varphi_{m}=\frac{2\pi \Delta l_{m}}{\lambda }$$

From this, the direction vector of the incident signal can be derived as follows:

(3) $$a(\theta_{k})={\left[1,e^{i2\pi \Delta \varphi_{2}}\cdots e^{i2\pi \Delta \varphi_{M}}\right]}^{T}$$

The signals received by a navigation signal receiver include many satellite signals and many interfering signals, in addition to noise. It is assumed that the signals and noise are independent of each other. Also, since the satellites are very far away from the navigation receiver, it is approximated to expect that the azimuth angle between the satellite signals and the antenna array does not change in a short period of time when the navigation receiver is in a highly dynamic scenario, but since the distance between the jamming source and the navigation receiver is relatively close to the receiver, it is assumed that the incoming direction of the jamming signals will change rapidly. Assume there is a satellite signal $s\left(t\right)$ (i.e., the desired signal) at a distant location, with a direction of arrival (DOA) of $\theta_{s}$. In addition, there are N jamming signals $i_{j}\left(t\right)$ (j=1,2⋯N) with their respective directions of arrival $\theta_{ij}$. The additive white noise received at each antenna element is denoted as $n_{k}(t)$ (with a mean of 0 and variance $\delta^{2}$). Under this assumption, the received signal at the k-th antenna element can be expressed as follows:

(4) $$x_{k}(t)=as_{k}(\theta_{s})s(t)+\sum_{j=1}^{N}as_{k}(\theta_{ij})i_{j}(t)+n_{k}(t)$$

Then, the signal received by the array is modeled as follows:

(5) $$\left[\begin{array}{c} x_{1}(t)\\ \vdots \\ x_{N+1}(t) \end{array}\right]=\left[\begin{array}{cccc} as(\theta_{s}) & as(\theta_{ij}) & \cdots & as(\theta_{iN}) \end{array}\right]\left[\begin{array}{c} s(t)\\ i_{1}(t)\\ \vdots \\ i_{N}(t) \end{array}\right]+\left[\begin{array}{c} n_{1}(t)\\ n_{2}(t)\\ \vdots \\ n_{N+1}(t) \end{array}\right]$$

It can be simplified as follows:

(6) $$x(t)=AS(t)+n(t)=as(\theta_{s})s(t)+\sum_{j=1}^{N}as(\theta_{ij})i_{j}(t)+n(t)$$

Table 1 shows the physical meaning of symbols in the signal model.

The covariance matrix of the received signal is mainly affected by interfering signals and noise because the satellite is far away from the receiver, and the received satellite signal strength is low. The covariance matrix of the received signal can be expressed as follows:

(7) $$R_{x}=E[x(t)x^{H}(t)]\approx \sum_{j=1}^{N}\delta_{j}^{2}as(\theta_{j})as^{H}(\theta_{j})+\delta_{n}^{2}I$$

In practical scenarios, adaptive beamforming requires the signal to be relatively stable. However, the covariance matrix in the equation is continuously changing. Therefore, the covariance matrix of the received signal is typically replaced by the sample covariance matrix of the received signal. Let K represent the number of snapshots of the beamformer (the larger the number of snapshots, the more information is available, and the better the algorithm’s performance). The sample covariance matrix of the received signal can be expressed as follows [13]:

(8) $$R=\frac{1}{K}E[\hat{x}(t){\hat{x}}^{H}(t)]$$

where $\hat{x}(t)$ represents the extraction of K sampling points from x.

The subsequent algorithms in this paper will focus on the sample covariance matrix.

The output SINR of the array is used as an evaluation metric for beamforming anti-jamming performance. The output SINR of the array can be derived from Equation (9).

(9) $$SINR=\frac{W_{opt}^{H}R_{s}W_{opt}}{W_{opt}^{H}R_{N+n}W_{opt}}$$

where $R_{s}$ is the sample covariance matrix of the desired satellite signal, and $R_{N+n}$ is the sample covariance matrix of the jamming signal and noise [14].

## 3. Theoretical Foundations of PI, CMT, and the Proposed Algorithm

### 3.1. PI Algorithm

When the power of the satellite signal is significantly lower than that of the jamming signal, adjustment is achieved by inverting the power ratio between the satellite and jamming signals [15]. This method minimizes the mean squared error between the reference signal and the array output as the objective function and adjusts the array weight vector based on system errors, thereby adaptively optimizing the weight vector. This algorithm does not distinguish between satellite and jamming signals. Its objective is to minimize the power of the array’s output signal. The stronger the encountered signal, the deeper the null is created, thereby suppressing both the satellite signal and the jamming signal at the input end.

As shown in Figure 1, it can be seen that there are M antenna array elements, and the PI selects the weights of each array element according to the Linearly Constrained Minimum Variance (LCMV) criterion so that the output power of the array is minimized. This is actually an adaptive beam-forming algorithm with constraints. Let the weight vector of the array be $W={\left[w_{1},w_{2},\cdots w_{M}\right]}^{T}$, the constraint of this algorithm be $W^{T}as_{0}=1$, and $as_{0}={\left[1,0,\cdots 0\right]}^{T}$; i.e., the weight vector should ensure that the gain of the first array element is 1 [16], so it can be assumed that the signal at the first array element of the array serves as the reference signal. The mathematical model of the LCMV criterion is given as follows:

(10) $$\left\{\begin{array}{c} \min P_{out}=E\left\{{\left|Y\right|}^{2}\right\}\\ s.t.W^{H}as_{0}=1 \end{array}\right.$$

In this paper, we assume that the satellite signal, jamming signal, and noise are statistically independent. Additionally, the noise in each channel is assumed to be Gaussian white noise. Based on these assumptions, we have the following:

(11) $$\begin{array}{ll} P_{out} & =E\left\{{\left|Y\right|}^{2}\right\}=E\left\{YY^{H}\right\}\\ & =E\left\{W^{H}XX^{H}W\right\}\\ & =W^{H}RW \end{array}$$

The optimal weight vector can be identified through the application of the LCMV criterion as follows:

(12) $$W_{opt}=R^{-1}as{\left(as^{H}R^{-1}as\right)}^{-1}$$

Thus, the output of the array after weighted summation can be expressed as follows:

(13) $$Y=W^{H}X$$

The nulls formed by the PI algorithm are very narrow, so the nulls formed in highly dynamic environments may not align well with the jamming directions. As a result, the algorithm is insufficient for jamming suppression in highly dynamic scenarios. Additionally, while suppressing jamming, this algorithm may also suppress the desired signal.

### 3.2. CMT Algorithm

The CMT algorithm sharpens the sample covariance matrix by applying a sharpening matrix $T_{CMT}$. It is assumed that near the jamming sources received by the receiver, there are K virtual jamming sources of equal strength arranged at specific spatial intervals, which are used to construct a new covariance matrix for null broadening. The new covariance matrix can be expressed as follows:

(14) $$\bar{R}=R⊗T_{CMT}$$

⊗ represents the Hadamard product, and the element in the m-th row and n-th column of the sharpening matrix $T_{CMT}$ is as follows:

(15) $$T_{mn}=\frac{\sin\left(\left(m-n\right)\Delta \right)}{\left(m-n\right)\Delta }=sinc\left(\sin\left(\frac{\left(m-n\right)\Delta }{\pi }\right)\right)$$

where Δ is the perturbation factor, the magnitude of which determines the width of the null after broadening [17].

Although the algorithm can broaden the null, it will also make the null shallow. Furthermore, the inhibition of jamming may simultaneously inhibit the useful signal, resulting in an ineffective anti-jamming effect.

### 3.3. Eigenvalue Sorting-Based Null Broadening Algorithm

To address the issues of the two aforementioned algorithms, this paper proposes a null broadening method based on eigenvalue sorting.

Its eigenvalue decomposition is obtained from the sample covariance matrix derived in Section 2.2:

(16) $$R=\sum_{k=1}^{N+1}\lambda_{k}u_{k}u_{k}^{H}$$

Therefore, we have $\lambda_{1}>\lambda_{2}>\lambda_{3}>\cdots >\lambda_{K}>\cdots >\lambda_{M}$, $\lambda_{K}$ is the eigenvalue of the covariance matrix (representing signal power), and $u_{k}$ is the eigenvector corresponding to the eigenvalue $\lambda_{K}$ (representing signal spatial distribution). The signal strengths decrease in the following order: jamming signals, noise, and finally, the satellite signal. In the matrix formed by the eigenvectors of the signals, the larger the eigenvalue corresponding to an eigenvector, the higher the signal power associated with that eigenvector. Therefore, the K largest eigenvalues and their corresponding eigenvectors can be selected to identify the jamming signals. (The degrees of freedom for an array of M elements is M−1; thus, the eigenvectors corresponding to the first M−1 largest eigenvalues can be selected to construct the interference subspace.) Based on this, the jamming signal subspace $E=\left\{u_{1,}u_{2,}u_{3,}\cdots u_{K}\right\}$ is constructed. Let the steering vector of the jamming signal be represented by a matrix $As=\left\{as_{1,}as_{2,}as_{3,}\cdots as_{K}\right\}$, and these two subspaces are considered to be equal.

(17) $$span\left\{u_{1,}u_{2,}u_{3,}\cdots u_{K}\right\}=span\left\{as_{1,}as_{2,}as_{3,}\cdots as_{K}\right\}$$

As mentioned before, the feature subspace corresponding to the interfering signal and the guiding vector subspace corresponding to the interfering signal are equal, so the feature subspace corresponding to the interfering signal can be used to find the projection matrix Q of the interfering signal subspace, and Q is obtained as follows:

(18) $$Q=E{\left(E^{H}E\right)}^{-1}E^{H}$$

Since the signals received by the navigation receiver’s RF antenna array are a mixture of desired navigation signals, jamming signals, and noise, a projection transformation is applied to separate the desired navigation signals from the jamming signals, filter out the noise, and ultimately obtain the optimized sample data.

(19) $$X_{1}(t)=(Q+I)X(t)$$

Subsequently, the updated sample covariance matrix is obtained as follows:

(20) $$R_{1}=E(X_{1}(t)X_{1}^{H}(t))=(Q+I)R{\left(Q+I\right)}^{H}$$

To improve the stability of the algorithm, a diagonal loading procedure is applied to the updated covariance matrix:

(21) $$R_{2}=R_{1}+\mu I$$

Here, μ represents the diagonal loading factor. The diagonal loading factor μ is usually chosen based on the noise power estimate of $\mu =10\delta_{n}^{2}$ (this is just an empirical value). I denotes the identity matrix with dimensions identical to those of $R_{1}$.

Since the number of snapshots K determines the quality of the signal, a larger number of snapshots leads to better beamforming performance. However, this also increases the complexity of the processing. To address this, forward–backward averaging is applied to the covariance matrix, effectively doubling the number of snapshots. This allows for improved beam broadening performance even when the number of snapshots is small.

(22) $$R_{3}=\frac{R_{2}+UR_{2}^{T}U}{2}$$

where the subdiagonal of the U-matrix is one, and all other elements are 0 matrices.

Then, the matrix $R_{3}$ is sharpened according to the CMT algorithm, and the sharpened matrix is substituted into the PI algorithm to obtain the optimal weight vector.

**Figure 2:**
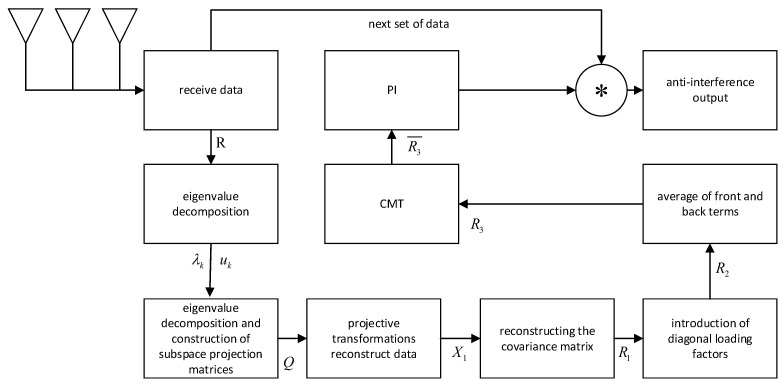
The flowchart of the proposed algorithm. Note: The ∗ in the figure represents multiplication.

Based on the above analysis, the steps of the proposed algorithm are as follows, and the algorithm flowchart is shown in Figure 2.

1. Perform eigenvalue decomposition on the covariance matrix of the received signal.

2. Sort the eigenvalues and construct the eigen-subspace projection matrix Q.

3. Apply a projection transformation to extract the jamming subspace and enhance the null.

4. Reconstruct the covariance matrix and add a diagonal loading factor, followed by averaging the before-and-after terms.

5. Sharpen the covariance matrix using the CMT algorithm, which enables null broadening.

6. Calculate the optimal weight vector using the PI algorithm to suppress jamming and output the jamming-suppressed signal.

### 3.4. Complexity Comparison and Analysis of the Algorithms

The complexities and core steps of the three algorithms are shown in Table 2:

M is the number of array elements, and K is the number of snapshots.

The PI algorithm has the lowest computational cost for actual computational complexity, while the CMT algorithm has a complexity similar to that of the PI algorithm but includes an additional Hadamard product operation. The proposed algorithm performs three $O\left(M^{3}\right)$ operations, resulting in the highest computational cost, and its computation time is maybe 2–3 times that of the PI algorithm. The proposed algorithm sacrifices higher computational costs for enhanced anti-jamming performance.

## 4. Performance Simulation Analysis

This experiment assumes two interfering signals with initial arrival angles of −40° and 50°, respectively. As the satellite is relatively far away from the receiver, it can be assumed that the arrival angle of the satellite signal remains unchanged over short periods of time when the receiver is in a highly dynamic environment. Based on the geometric model $w=\frac{v}{d}*\frac{180^{\text{°}}}{\pi }$, assuming the carrier velocity (receiver speed) is v=500 m/s and the jamming source distance is d≈14.3km, the angular velocity $w=2^{\circ }/s$ can be calculated. The distance d≈14.3km is within the typical engagement range in hypersonic weapons or anti-missile scenarios. The arrival angle of the satellite signal is set to 0°. When the receiver is in a highly dynamic scene, it is assumed that the incoming interfering signal changes by 2° per second, and 2 s of simulation data is used as the object of study, so the jamming range of the interfering signal is set to −40~−44° and 50~54°. The experiments were carried out using linear uniform arrays, and arrays of 3, 6, 9, and 12 array elements were studied separately. Simulated GPS L5 satellite signals are used, with an IF of 15.48 MHz, a sampling rate of 4 MHz, a Doppler frequency of 50,000 Hz, and a code phase of 5000 chips. The array element spacing is set to half the wavelength of the satellite signal’s carrier frequency. The jamming signal frequencies are 1175.4 MHz and 1177.4 MHz, with an SNR of -25 dB and an ISR fixed at 90 dB. The satellite’s PRN is set to 1, and the diagonal loading factor is 10.

### 4.1. Comparison and Analysis of Three Algorithms

**Figure 3:**
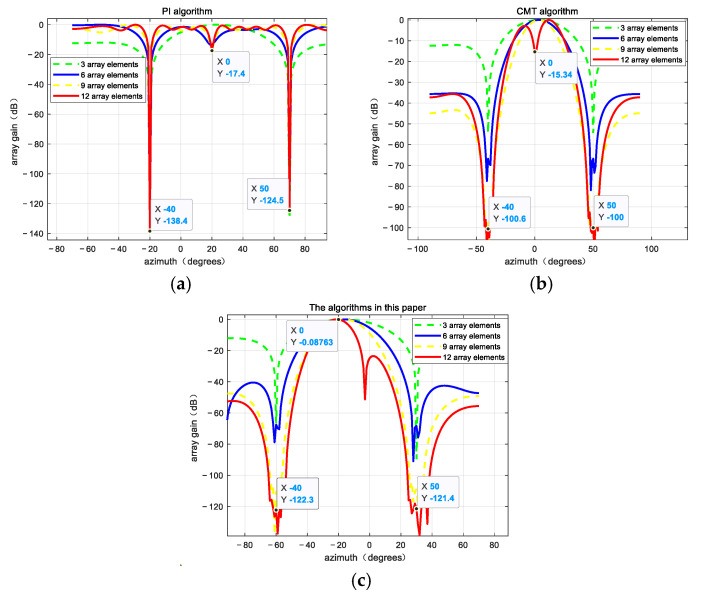
Beams are formed by the three algorithms. (**a**) PI algorithm; (**b**) CMT algorithm; (**c**) proposed algorithm.

The data points from the first 0.5 s are used as input signals for analysis, assuming that the arrival angles of the jamming signals remain unchanged during this period and are equal to their initial directions. The experimental results are shown in Figure 3.

For the PI algorithm, when the navigation receiver remains in a stable state, the jamming signals from the directions of −40° and 50° will be significantly suppressed. However, due to the narrowness of the nulls, the algorithm becomes unsuitable in highly dynamic environments, leading to a significant decline in its ability to suppress dynamic jamming. Although the CMT algorithm can generate wider nulls in the direction of jamming, the depth of the nulls decreases, and it also suppresses the desired signals to some extent. As can be seen from Figure 3, the algorithm in this paper can form a deeper null out of the jamming coming direction, and the null has a more obvious broadening and can also protect the useful signal. In addition, in the uniform line array, the beam broadening effect is best at 12 array elements, and the input ISR is 90 dB; at this time, the average deepening of the null of the jamming coming out is about 22 dB, and secondly, the gain of the useful signal coming out is about 15dB, so this algorithm is very suitable for navigation receivers in highly dynamic scenarios. Table 3 presents the null depth formed by different algorithms under varying numbers of antenna array elements, along with the gain in the direction of the useful signal.

### 4.2. Comparison and Analysis of Acquisition Performance in Highly Dynamic Environments

A total of 2 s of data was simulated, and the data from 0 to 2 s were analyzed separately. Table 4 shows the incoming jamming for 0~2 s.

A new mathematical model has been established to analyze the acquisition performance in highly dynamic scenarios. The N sampling points within 0~2 s are divided into three segments: In the first segment, the jamming directions are −40° and 50°; in the second segment, the jamming directions are −42° and −52°; and in the third segment, the jamming directions are −44° and 54°. The first segment of $\frac{N}{3}$ data points is used to calculate the optimal weight vector, with a uniform linear array consisting of 12 elements. The obtained weight vector is then applied to process the subsequent two segments of $\frac{N}{3}$ data points, resulting in the array’s output signals.

(23) $$Y_{2}=W_{1}^{H}X_{2}$$

(24) $$Y_{3}=W_{1}^{H}X_{3}$$

Then, the array output signal is acquired. To simplify the experiment, the $Y_{3}$ signal is used for acquisition.

**Figure 4:**
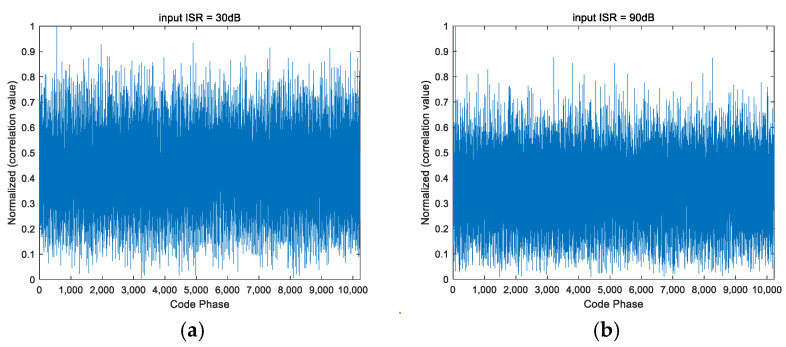
Signal acquisition performance after PI processing under different ISRs. (**a**) ISR = 30 dB; (**b**) ISR = 90 dB.

**Figure 5:**
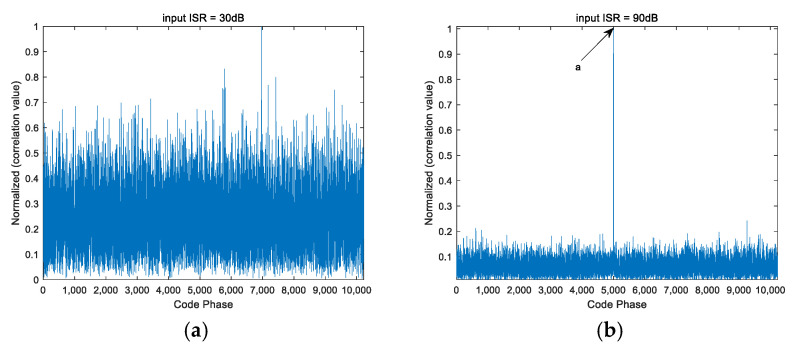
Signal acquisition performance after CMT processing under different ISRs. (**a**) ISR = 30 dB; (**b**) ISR = 90 dB.

**Figure 6:**
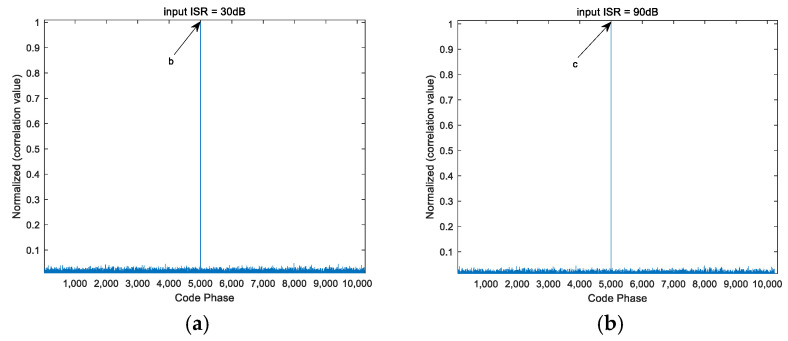
Signal acquisition performance after processing with the proposed algorithm under different ISRs. (**a**) ISR = 30 dB; (**b**) ISR = 90 dB.

From Figure 4, Figure 5 and Figure 6, it can be seen that the PI algorithm is not suitable for spatial anti-jamming in highly dynamic scenarios, as it causes jamming signals to overshadow the desired satellite signals, resulting in failure to acquire the satellite signal. On the other hand, both the CMT algorithm and the beam broadening algorithm proposed in this paper can be applied to spatial anti-jamming in highly dynamic environments. When the input ISR is large, the acquired code phase is correct. However, when the input ISR is small, the CMT beam broadening algorithm produces errors in the acquisition results. Compared to the CMT nulls broadening algorithm, the method proposed in this paper performs better.

Note: In Figure 5 and Figure 6, the coordinates of points a, b, and c are the highest points of the line segments indicated by the arrows. The coordinates of a, b, and c are all (5000, 1).

### 4.3. Comparison and Analysis of Algorithm Performance with Different Snapshot Numbers

**Figure 7:**
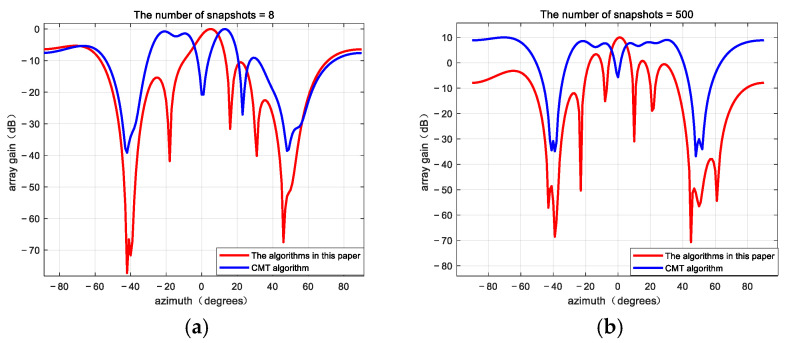
A beam is formed of the two algorithms using different snapshot numbers. (**a**) The number of snapshots is 8; (**b**) the number of snapshots is 500.

**Figure 8:**
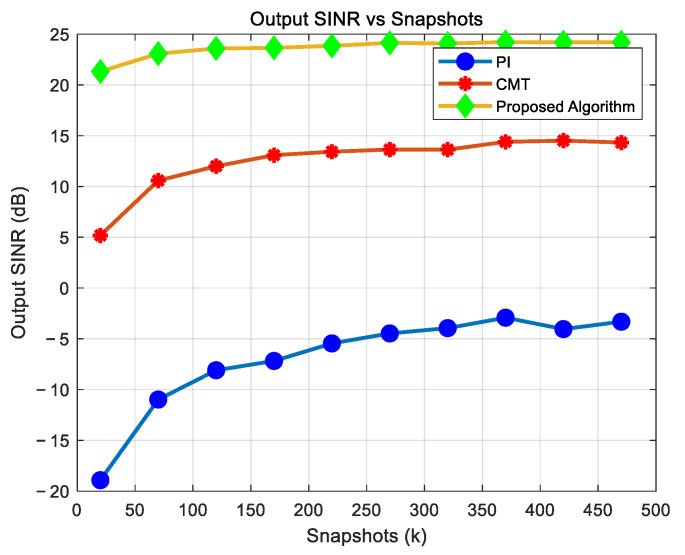
Output SINR of various algorithms at different numbers of snapshots.

To evaluate the impact of snapshot numbers on algorithm performance, beam patterns of the proposed algorithm and the CMT null broadening algorithm were analyzed under the conditions of 500 and 8 snapshots, an input ISR of 30 dB, an SNR of 5 dB, and 12 array elements, as illustrated in Figure 7. Using the mathematical model introduced in Section 4.2, Monte Carlo simulations were performed to compare the output SINR of several algorithms under different snapshot numbers, with a 2° deviation in the jamming direction. The Monte Carlo simulations were repeated 50 times, and the results are shown in Figure 8.

As shown in Table 5, when the number of snapshots is large, both algorithms can broaden the nulls in the direction of jamming, and the algorithm proposed in this paper outperforms the CMT algorithm, with deeper nulls in the jamming direction and higher gain in the desired signal direction. When the number of snapshots is small, the proposed algorithm can still broaden the nulls in the jamming direction while maintaining a relatively deep null. In contrast, when the number of snapshots is small, the CMT algorithm shows poor null broadening performance in the jamming direction, with the null depth becoming shallow and the negative gain in the desired signal direction increasing significantly. As shown in Figure 8, the PI algorithm exhibits very low output SINR regardless of whether the number of snapshots is small or large, which aligns with theoretical expectations. This is because the PI algorithm cannot suppress jamming when there is a deviation in the jamming direction. When the number of snapshots is small, the output SINR of the CMT algorithm is significantly lower than that of the proposed algorithm. Moreover, even as the number of snapshots increases, the CMT algorithm’s output SINR consistently remains below that of the proposed algorithm.

Thus, in scenarios with a small number of snapshots, the PI algorithm fails to suppress jamming signals in highly dynamic environments and cannot protect the useful signals from suppression. Similarly, the CMT algorithms also fail to protect useful signals and exhibit inferior anti-jamming capabilities compared to the proposed algorithm. In contrast, the proposed algorithm maintains superior performance even with a limited number of snapshots.

### 4.4. Analysis of the Impact of SNR on Algorithm Performance in Highly Dynamic Scenarios

A Monte Carlo simulation was conducted to compare the output SINR of several algorithms under different SNR conditions. To simulate highly dynamic scenarios, the mathematical model introduced in Section 4.2 was utilized (jamming direction deviation of 2°). The simulation was repeated 50 times.

**Figure 9:**
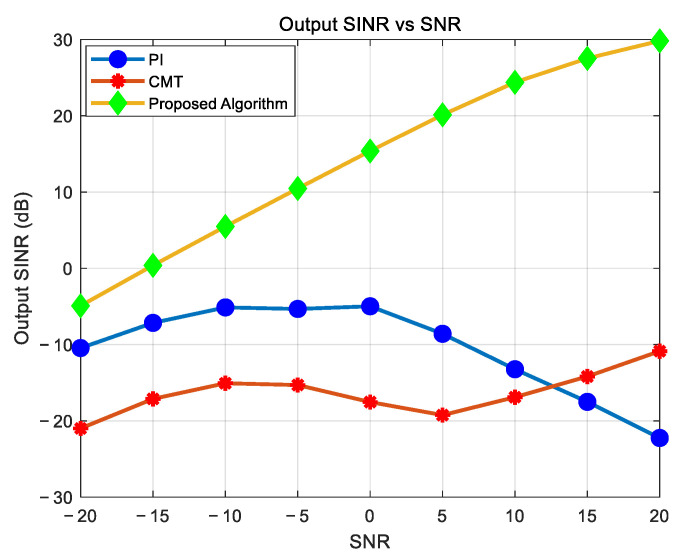
Relationship between input SNR and output SINR.

From Figure 9, the experimental results demonstrate that when the input SNR is low, the output SINR of both the PI and CMT algorithms remains very low, rendering these algorithms ineffective. As the input SNR gradually increases, the output SINR of the CMT algorithm also increases. However, due to the extremely narrow nulls produced by the PI algorithm, it fails to suppress jamming in highly dynamic scenarios. Consequently, even with increasing input SNR, the output SINR of the PI algorithm remains very low and may even exhibit a downward trend. In contrast, the proposed algorithm achieves a near-linear relationship between the input SNR and output SINR. This demonstrates its significantly superior performance compared to the other algorithms, particularly in highly dynamic scenarios.

### 4.5. Evaluation of Algorithm Performance with Jamming Direction Deviation

Under the conditions of 500 snapshots, an SNR of 5 dB, and an ISR of 30 dB, Monte Carlo simulations were conducted to compare the output SINR of the proposed algorithm with two other algorithms under varying jamming direction deviations. The number of Monte Carlo iterations was set to 50.

**Figure 10:**
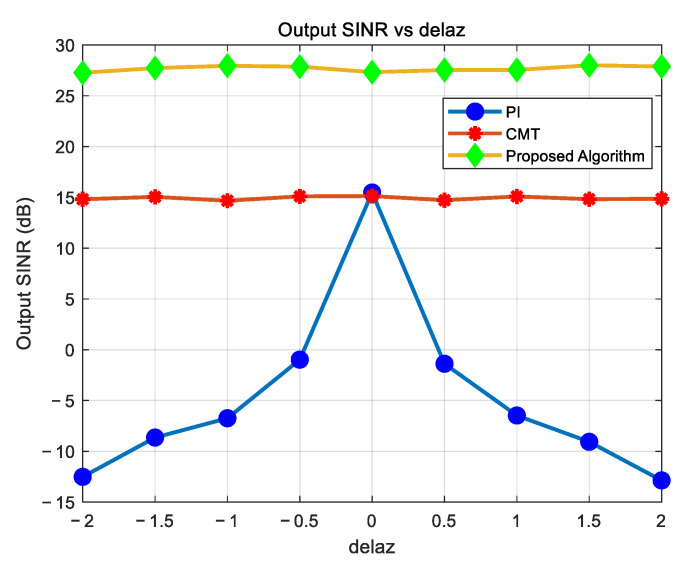
Variation in output SINR with jamming direction angle deviation.

As shown in Figure 10, when there is a deviation in the jamming direction, the PI algorithms fail to suppress jamming, which is consistent with theoretical expectations. Both the CMT algorithm and the proposed algorithm maintain relatively stable output SINR even with a 4° angle deviation, with the proposed algorithm consistently outperforming the CMT algorithm in terms of output SINR.

### 4.6. Comparison of Algorithm Performance Under Different Interference Types

**Figure 11:**
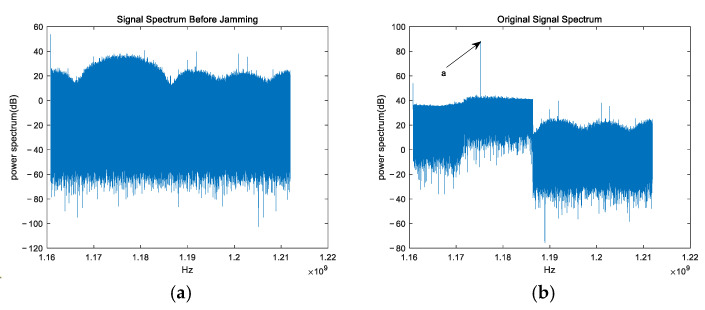
Frequency spectrum of the input signal. (**a**) Frequency spectrum before jamming injection; (**b**) frequency spectrum after injection of wideband and narrowband jamming.

Using the GPS L5 satellite signal from the simulation in Section 4 as the input, narrowband jamming with a frequency of 1175 MHz and wideband jamming with a bandwidth of 8 MHz (jamming direction angle deviation is still 2°), both with an SNR of 10 dB and an ISR of 30 dB, are introduced. The frequency spectra before and after the jamming injection are shown in Figure 11.

**Figure 12:**
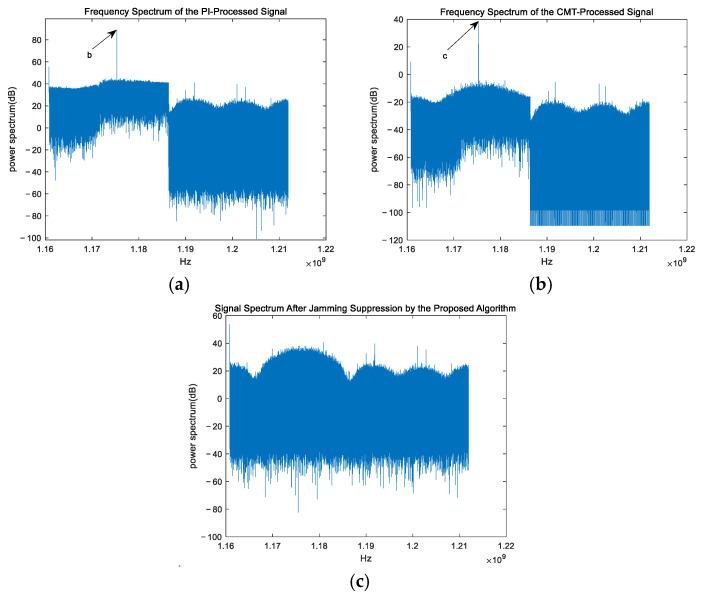
Frequency spectra of output signals after jamming suppression by three algorithms. (**a**) PI algorithm; (**b**) CMT algorithm; (**c**) proposed algorithm.

Next, the frequency spectra of the signals after jamming suppression by three algorithms are analyzed. Figure 12 shows the frequency spectra of the output signals after jamming suppression by three algorithms, where (a), (b), and (c) correspond to the results of the three algorithms, respectively.

Note: In Figure 11 and Figure 12, the coordinates of points a, b, and c are the highest points of the line segments indicated by the arrows. The coordinates of a, b, and c are (1,175,000,000, 88.11), (1,175,000,000, 88.29), and (1,175,000,000, 38.47), respectively.

From Figure 12a, it can be observed that the PI algorithm shows almost no jamming suppression capability when there is a deviation in the jamming angle, which is consistent with the theoretical expectations. From Figure 12b, it can be observed that the CMT algorithm can suppress narrowband and wideband jamming to some extent when there is a deviation in the jamming angle. However, it does not completely eliminate the jamming, and the output signal frequency is also reduced. This indicates that the null in the direction of the jamming has been broadened, but the depth of the null remains shallow, while the negative gain is generated in the direction of the satellite signal. From Figure 12c, it can be observed that the proposed algorithm is able to almost eliminate both wideband and narrowband jamming even when there is a deviation in the jamming angle, while the satellite signal is almost unaffected. This indicates that the null in the direction of the jamming has been broadened, the depth of the null is sufficiently deep, and there is almost no negative gain generated in the direction of the satellite signal. It is evident that, in highly dynamic scenarios, the proposed algorithm significantly suppresses both wideband and narrowband interference, while also greatly improving the output quality of the satellite signal.

## 5. Discussion

In highly dynamic scenarios, the DOA of jamming sources changes rapidly. The PI algorithm struggles to adapt to these fast-changing jamming directions due to its non-real-time weight updates and narrow nulls. Consequently, jamming signals can easily fall outside the null regions created by the PI algorithm, leading to its failure. Although the CMT algorithm effectively broadens nulls, thereby improving anti-jamming performance to some extent, the null broadening comes at the expense of reduced null depth. This limitation hinders the improvement of output SINR, leaving the performance of the CMT algorithm less than satisfactory.

This paper proposes a novel null broadening method that enhances the jamming signal’s weight by sorting the eigenvalues of the sampled covariance matrix and reconstructing the covariance matrix to form deeper nulls. Integrating the CMT algorithm for null broadening effectively improves anti-jamming capability in highly dynamic environments and significantly enhances the output SINR.

However, this study primarily focuses on linear arrays, while circular and planar arrays have been widely adopted in modern highly dynamic platforms due to their superior spatial distribution and beamforming capabilities. Validating the applicability of the proposed algorithm in circular and planar arrays is an important direction for future research. Additionally, the algorithm discussed in this paper considers only the case of two fixed jamming sources. However, in complex highly dynamic scenarios, multiple fixed or moving jamming sources may exist simultaneously, posing greater challenges to the algorithm’s effectiveness and performance. Furthermore, when the direction of the jamming is close to the direction of the satellite signal, the algorithm significantly suppresses the satellite signal, leading to the loss of lock in the subsequent tracking loop. Future research will further explore the performance of this algorithm in environments with multiple fixed or moving jamming sources, and investigate a new approach to address the algorithm’s failure when the direction of the jamming is close to the direction of the satellite signal. Building on this, the algorithm’s performance in circular, and square array applications will be verified through simulation. In the future, the algorithm can be deployed on a hardware platform to verify its feasibility in real-world scenarios. These studies aim to enhance the algorithm’s robustness and practicality in highly dynamic scenarios.

## Figures and Tables

**Table 1:** Physical meanings of the symbols in the signal model.

Symbol	Physical Meaning
$as_{k}(\theta_{s})$	Phase Difference of the Satellite Signal at the *k*-th Array Element Relative to the Reference Element
$as(\theta_{s})$	Guiding Vector of the Satellite Signal
$as_{k}(\theta_{ij})$	Phase Difference of the *j*-th Jamming Signal at the *k*-th Array Element Relative to the Reference
$as(\theta_{ij})$	Guiding Vector of the *j*-th Jamming Signal
s(t)	Satellite Signal
$i_{j}(t)$	*j*-th Jamming Signal
S(t)	Satellite Signal and Jamming Signal
A	Guiding Vector Matrix
n(t)	Noise in the Input Signal

**Table 2:** Complexity and core steps of the three algorithms.

Algorithms	Theoretical Time Complexity	Core Steps
PI algorithm	$O\left(M^{3}\right)$	$\mathrm{Covariance}\;\mathrm{Matrix}\;\mathrm{Estimation}\;(O\left(KM^{2}\right)$$)+\mathrm{Matrix}\;\mathrm{Inversion}\;(O\left(M^{3}\right)$)
CMT algorithm	$O\left(M^{3}\right)$	$\mathrm{PI}\;\mathrm{Steps}+\mathrm{Hadamard}\;\mathrm{Product}\;(O\left(M^{2}\right)$)
Proposed Algorithm	$O\left(M^{3}\right)$	$\mathrm{PI}\;\mathrm{Steps}+\mathrm{Eigenvalue}\;\mathrm{Decomposition}\;(O\left(M^{3}\right)$$)+\mathrm{Subspace}\;\mathrm{Projection}\;(O\left(M^{3}\right)$) + CMT Sharpening

**Table 3:** Null depth and useful signal gain for different numbers of array elements.

Number of Array Elements	PI	CMT	The Algorithm Proposed in this Paper
3	–0.1003	–0.1004	–0.09977
	–125.8	–54.39	–67.405
6	–13.4	–0.2393	–0.2226
	–128.9	–66.8	–67.92
9	–15.96	–0.4704	–0.3966
	–128.9	–105.85	–115.5
12	–17.4	–15.34	–0.08763
	–131.45	–100.3	–121.85

Note: The first value in each cell represents the gain in the direction of the signal, while the second value represents the null depth in the direction of the jamming. The units of these values are in dB.

**Table 4:** Direction of jamming signals from 0 to 2 s.

Time	0 s	1 s	2 s
Direction of Jamming 1	–40°	–42°	–44°
Direction of Jamming 2	50°	52°	54°

**Table 5:** Null depth and useful signal gain of the algorithm under different numbers of snapshots.

Number of Snapshots	CMT	The Algorithm Proposed in this Paper
8	–20.79	–1.933
	–34.66	–61.285
500	–15.69	–0.1937
	–40.48	–65.635

Note: The first value in each cell represents the gain in the direction of the signal, while the second value represents the null depth in the direction of the jamming. The units of these values are in dB.

## Data Availability

Data are contained within the article.
